# Automated and accurate assessment for postural abnormalities in patients with Parkinson’s disease based on Kinect and machine learning

**DOI:** 10.1186/s12984-021-00959-4

**Published:** 2021-12-04

**Authors:** Zhuoyu Zhang, Ronghua Hong, Ao Lin, Xiaoyun Su, Yue Jin, Yichen Gao, Kangwen Peng, Yudi Li, Tianyu Zhang, Hongping Zhi, Qiang Guan, LingJing Jin

**Affiliations:** 1 Neurological Department of Tongji Hospital, Tongji University School of Medicine, Shanghai, China; 2 IFLYTEK Suzhou Research Institute, E4, Artificial Intelligence Industrial Park, Suzhou Industrial Park, Suzhou, China; 3 Department of Neurorehabilitation, Yangzhi Rehabilitation Hospital (Shanghai Sunshine Rehabilitation Center), Tongji University School of Medicine, Shanghai, China

**Keywords:** Parkinson’s disease, Postural abnormalities, Kinect, Machine learning

## Abstract

**Background:**

Automated and accurate assessment for postural abnormalities is necessary to monitor the clinical progress of Parkinson’s disease (PD). The combination of depth camera and machine learning makes this purpose possible.

**Methods:**

Kinect was used to collect the postural images from 70 PD patients. The collected images were processed to extract three-dimensional body joints, which were then converted to two-dimensional body joints to obtain eight quantified coronal and sagittal features (F1-F8) of the trunk. The decision tree classifier was carried out over a data set established by the collected features and the corresponding doctors’ MDS-UPDRS-III 3.13 (the 13th item of the third part of Movement Disorder Society-Sponsored Revision of the Unified Parkinson’s Disease Rating Scale) scores. An objective function was implanted to further improve the human–machine consistency.

**Results:**

The automated grading of postural abnormalities for PD patients was realized with only six selected features. The intraclass correlation coefficient (ICC) between the machine’s and doctors’ score was 0.940 (95%CI, 0.905–0.962), meaning the machine was highly consistent with the doctors’ judgement. Besides, the decision tree classifier performed outstandingly, reaching 90.0% of accuracy, 95.7% of specificity and 89.1% of sensitivity in rating postural severity.

**Conclusions:**

We developed an intelligent evaluation system to provide accurate and automated assessment of trunk postural abnormalities in PD patients. This study demonstrates the practicability of our proposed method in the clinical scenario to help making the medical decision about PD.

## Background

Parkinson’s disease (PD) is the second most common chronic neurodegenerative disease after Alzheimer’s disease, characterized by motor impairments with tremor, rigidity and akinesia/bradykinesia as cardinal symptoms [1]. Postural abnormalities are frequent and disabling complications of PD. A cross-sectional study involving 811 PD patients showed the prevalence of postural abnormalities reached 21.5% [2]. Common postural abnormalities in PD include sagittal abnormalities: camptocormia and anterocollis; coronal abnormalities: Pisa syndrome and scoliosis [3]. Abnormal postures cause pain and balance dysfunction, aggravating walking difficulties with important impacts on life quality [2, 4]. At present, its pathogenesis is unclear and there is no recognized objective and quantitative assessment method. Early recognition and standardized management will be helpful to delay the progression of postural abnormalities in PD to avoid worse outcomes.

There are various manual methods for evaluating abnormal posture of PD, including clinical scales, inclinometer, wall goniometer and photo-based measurement [5–8]. The most commonly used scale for evaluating abnormal posture of PD is the 13th item of the third part of Movement Disorder Society-Sponsored Revision of the Unified Parkinson’s Disease Rating Scale (MDS-UPDRS-III 3.13), which is categorized into a discrete scale of five classes of increasing severity [9]. However, this method mainly relies on doctors’ subjective judgement, which brings obvious intra and inter-rater variability. Though the inclinometer and wall goniometer methods are inexpensive and easy to operate, they only provide limited features and the results are not accurate [5, 8, 10]. The photo-based measurement method is considered as the gold standard to measure postural abnormalities in PD [7]. Nevertheless, it is time-consuming and the reference anatomical sites are not consistent among different tests.

Device-assisted posture evaluation which adopts accelerometers, gyroscopes, and optoelectronic system for analysis, provides sensitive, objective and real-time assessments [11–13]. However, they require sensors with satisfactory sensitivity and stability, which can be expensive [14]. Besides, wearable sensors are often not user-friendly. Patient engagement with wearable sensors is modest, possibly due to the absence of meaningful feedback and troublesome wearing [15]. Moreover, the analytical methods are complicated [16]. Clinical and technical expertise are needed to eliminate ‘clinical noise’ in the data analysis. Systems that are based on depth camera might represent a valid solution to overcome both the high cost and encumbrance of wearable devices.

Kinect is a low-cost and non-contact tracking device developed by Microsoft [17]. Okada et al. adopted the Kinect-based system for in-home posture evaluation and visual feedback training [18]. However, the system was unable to assess the postural abnormality automatically. In the last decade, machine learning (ML) has been widely used in the medical field to help doctors in medical judgment and decision [19–23]. ML techniques have been used and compared for PD classification with Kinect depth cameras [24–26]. Several trials using Kinect for automated assessment of UPDRS limb tasks and postural instability have been reported with an accuracy ranging from 74.0% to 95% [25, 27–29], including finger tapping, fists opening and closing, pronation and supination, stomping, posture and standing. Ferraris et al. [27] analyzed the posture of 28 PD patients with a Kinect camera. They performed automated machine grading based on supervised classifier training and compared with the scores of two doctors. The intraclass correlation coefficient (ICC) between the two doctors’ scores was 0.77 while the ICC between the machine and the doctor’s grading was 0.74 which suggested the possibility of automated grading by the machine, but the performance was not satisfactory enough.

We tried to develop an intelligent system based on Kinect and machine learning for evaluating postural characteristics in PD patients and achieved accurate description and automated grading of postural abnormalities. Taking advantage of a larger data set, we introduced multi-dimensional features of trunk posture and used decision tree of supervised classifier with an objective function implanted to improve the human–machine consistency.

## Methods

### Experimental subjects

Consecutive patients diagnosed with PD in Tongji Hospital Affiliated to Tongji University from October 2018 to January 2020 were enrolled. The inclusion criteria were: (1) Meet the diagnostic criteria of clinically confirmed PD in the 2015 MDS Parkinson’s disease clinical diagnostic criteria [9]; (2) Able to stand by oneself. The exclusion criteria were: (1) Suspected or diagnosed with Parkinson’s superimposed syndrome or secondary Parkinson’s syndrome; (2) Patients with deformities or injuries of lower limbs; (3) Patients with mental disorders or cognitive impairments.

Written informed consent was obtained from all participants, and the present study was approved by the Ethics Committee of Shanghai Tongji Hospital.

### Devices and experimental procedure

In cooperation with iFLYTEK Suzhou Research Institute, a motion analysis device integrating a Kinect v2.0 depth camera (RGB 1920 × 1080 pixels @30fps, depth camera 512 × 424 pixels @30fps, 4-microphone linear phased array, Microsoft) was built. The Kinect camera was connected to an independent computer that ran a data capture program that saved the data generated during the recording process.

**Fig. 1:**
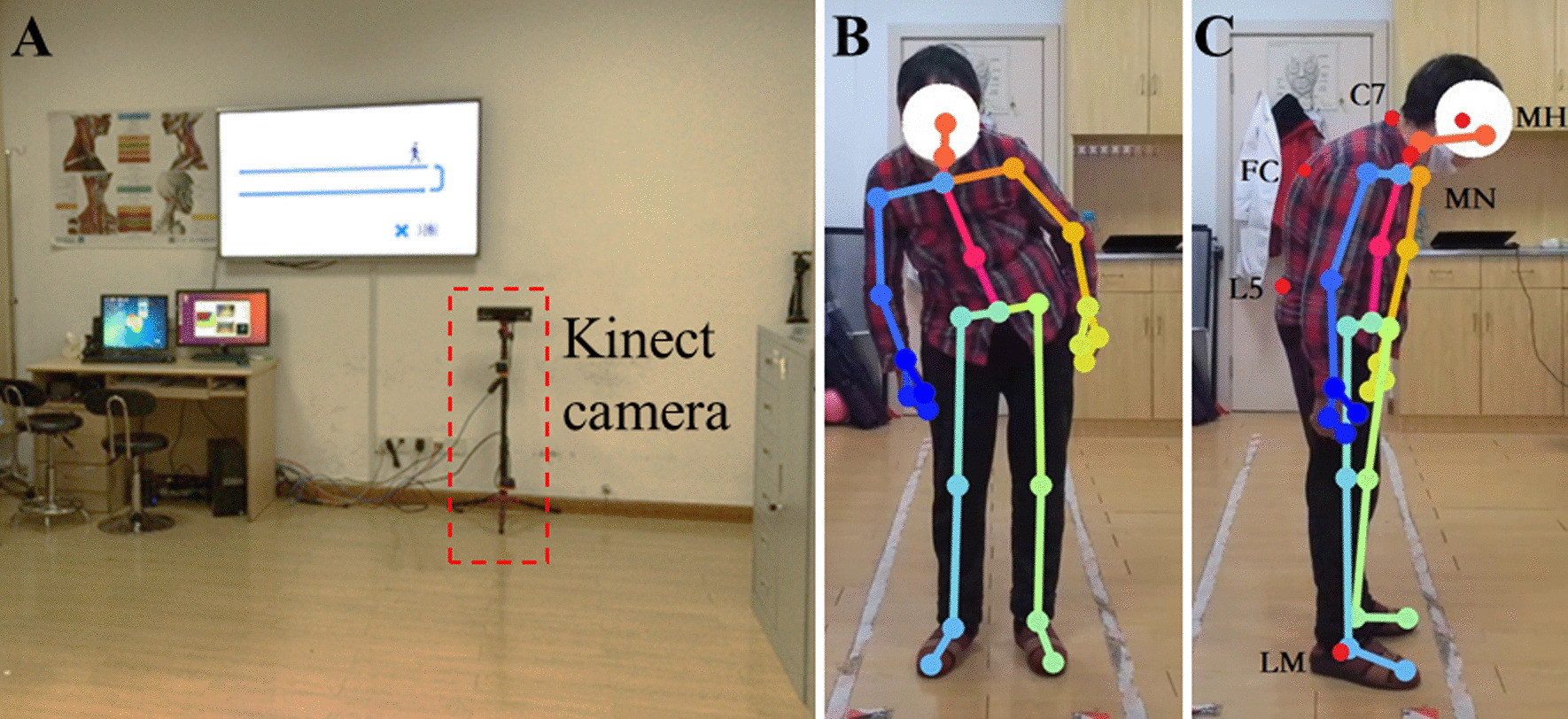
Real image showing the test scene, in which you can see the Kinect camera (**A**) and a participant undergoing the test (**B** and **C**)

The recording process was as follows (Fig. 1): Patients were asked to stand directly in front of the Kinect camera at ease for 5 s and then actively correct their abnormal posture for 5 s. After that, they were asked to turn left for 90°, relax and stand for 5 s, and then actively correct their abnormal posture for another 5 s. Patients were advised to wear close-fitting clothing and long hair should be tied up. Their gender, age, onset age, length of disease and Hoehn-Yahr scale were collected and recorded. Meanwhile, 2 professionally trained doctors (namely doctor1 and doctor2) individually rated the postural abnormalities of PD patients according to MDS-UPDRS-III 3.13.

### Data collection and processing

**Fig. 2:**
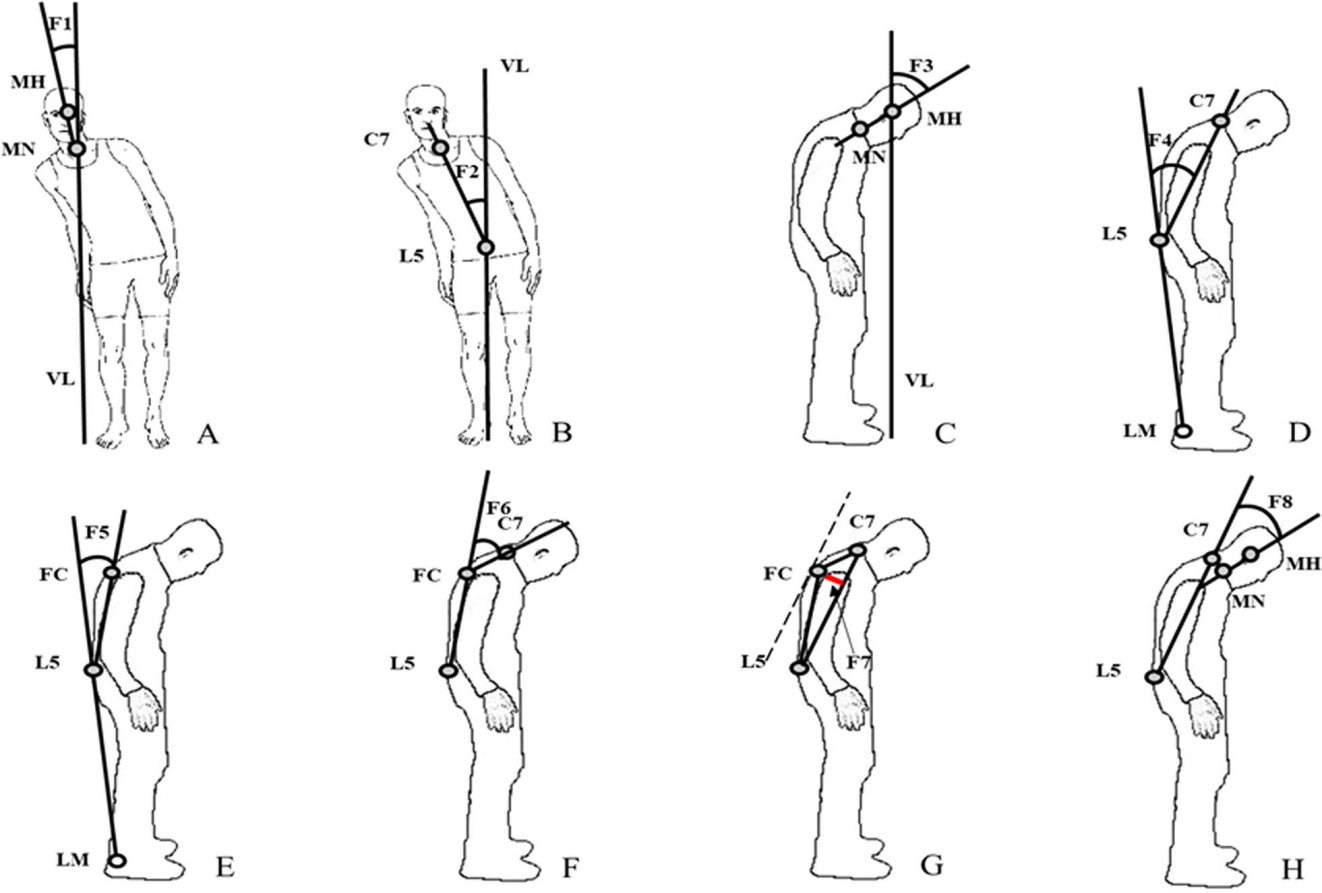
Illustration of the measured features for abnormal trunk posture (**A** and **B** were modified from [4])

**Fig. 3:**
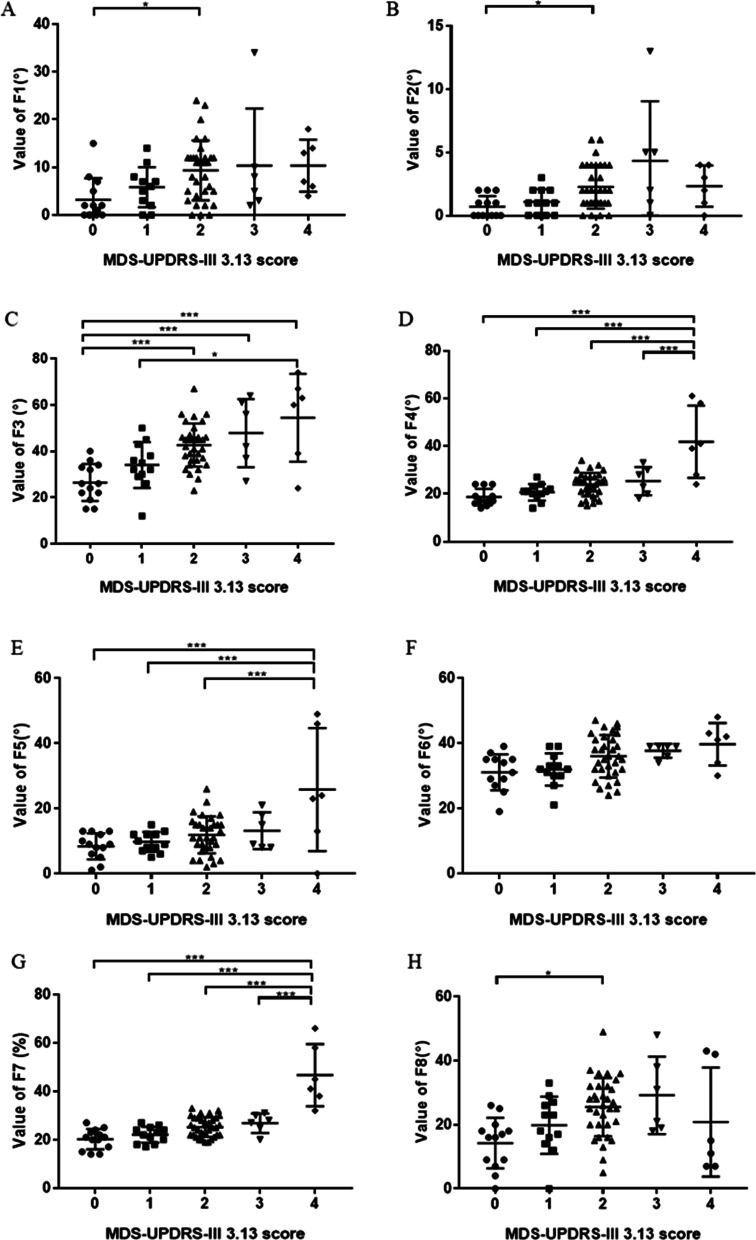
Intergroup comparison of postural features among groups of different MDS-UPDRS-III 3.13 score

According to previous reports combining clinical experience on the assessment methods of postural abnormalities, the collected abnormal posture features were defined as follows [7, 30, 31]: (1) F1:lateral flexion angle of head, the angle between the connecting line of midpoint of head (MH) and midpoint of neck (MN) on the coronal plane and the vertical line of the ground (VL) (Fig. 2A); (2) F2: lateral flexion angle of trunk, the angle between the connecting line of the 7th cervical spinous process (C7) and the 5th lumbar spinous process (L5) on the coronal plane and VL (Fig. 2B); (3) F3: forward flexion angle of head, the angle between the connecting line of MH and MN on the sagittal plane and VL (Fig. 2C); (4) F4: total forward flexion angle of trunk, the angle between the connecting line of L5 and lateral malleolus (LM) and the connecting line of C7 and L5 on the sagittal plane (Fig. 2D); (5) F5: forward flexion angle of trunk at the waist, the angle between the connecting line of L5 and LM and the connecting line of vertebral fulcrum (FC, which indicates the most convex point of the vertebra) and L5 on the sagittal plane (Fig. 2E); (6) F6: forward flexion angle of trunk at the thorax, the angle between the connecting line of FC and L5 and the connecting line of C7 and FC on the sagittal plane (Fig. 2F); (7) F7: distance between the most convex point of the back and the trunk axis, the pixel distance between FC and the connecting line of C7 and L5 on the sagittal plane(Fig. 2G); (8) F8: forward flexion angle of head relative to trunk, the angle between the connecting line of MH and MN and the connecting line of C7 and L5 on the sagittal plane (Fig. 2H).

Firstly, the Kinect depth map was used to segment the body part with the segmentation algorithm, which was conducive to the extraction of key points involved in features F1 to F8. The segmentation algorithm was set as follows:

$$d_{body} (x,y) = \left\{ \begin{gathered} 0\;\;\;\;\;\;\;\;\;if\;abs(d(x,y) - d_{c} ) < thr, \hfill \\ 1\;\;\;\;\;\;\;\;\;else \hfill \\ \end{gathered} \right.$$

$d_{body}$ denoted the segmented body depth map, in which 0 was background, 1 was body part. $d$ represented the depth of a map. $abs(d(x,y) - d_{c} )$ denoted the depth distance between the point $(x,y)$ and body center. $thr$ was the threshold.

Then, the depth images collected by the Kinect camera were processed frame by frame, and a skeleton composed of 25 three-dimensional(3D) body joints was extracted using human skeleton and muscle model [24, 27]. Next, the 3D body joints were further projected to the two-dimensional(2D) body joints on a plane figure. Since these extracted joints were inconsistent with the key points actually used for feature calculation, the initial joints of Kinect skeleton were used as the basis to further calculate the exact bone points for posture evaluation from a side view of the participant. Specifically, the head, neck and hip 2D joints of the Kinect skeleton were taken as benchmarks: we further segmented the segmented human body based on the morphological method by combining the coordinates of head and neck on the depth map to obtain the point MH; taking the 2D coordinates of neck and hip on the depth map as the initial points, we got the contour points C7 and L5 along the back based on the depth point cloud. After that, we got FC by taking C7 and L5 as the benchmarks and combining the points on the entire back resulted from human body segmentation. In this way, a total of 30 2D points were acquired. Then, the pixel distance between any two of the points or the angle between any two lines connecting the points was automatically calculated to obtain the quantified kinematic features of the posture F1 to F8. Particularly, the feature F7 was normalized according to the height of the human body, eliminating the effects of height differences. After that, a supervised learning method named decision tree was carried out over a data set established by the collected features and the corresponding doctors’ MDS-UPDRS-III 3.13 scores. An objective function was implanted to further improve the human–machine consistency. The objective function for minimizing calculation errors was set as follows:

$$\begin{gathered} f = mean(abs(doc - machine)) + \hfill \\ \begin{array}{*{20}c} {} & {} \\ \end{array} \lambda *len(find(abs(doc - machine) > 1)) \hfill \\ \end{gathered}$$

The objective function *f* consists of two parts. The first part is the deviation between the machine’s and the doctor’s score. The second part is the combination of the coefficient λ and cases number, which error between the machine’s and the doctors’ score exceeds 1. Firstly, using Gini index as the segmentation criterion, a set of sub-optimal trees were trained. Then, the grid search was conducted to search for the optimal decision tree depth and split strategy (‘best’ or ‘random’) in the sub-optimal trees. In other words, the optimal decision tree is the smallest difference (not more than 1 as much as possible) between the machine’s and the doctor’s scores. Finally, 5-fold cross-validation was performed on the sample set with expert annotations to avoid overfitting.

Metrics for evaluating machine learning performance such as accuracy, sensitivity, and specificity were obtained by applying the 5-fold cross-validation method for the decision tree. We firstly transferred the 5-class classification problem into five 2-class classification problems. The metrics for each 2-class classifier were calculated respectively. Finally, the acquired metrics were averaged to estimate the metrics for the 5-class classifier.

### Statistical analysis

Quantitative data were shown as mean ± Standard Deviation (SD). The normality of distribution of demographic, clinical data and F1 to F8 were initially tested using the Kolmogorov–Smirnov test. The corresponding difference of the related data among the five different groups based on the results of MDS-UPDRS-III 3.13 score were analyzed using one-way analysis of variance (ANOVA) initially when the data followed normal distribution. Otherwise, Kruskal–Wallis H-test was adopted. Spearman correlation analysis was conducted to establish correlation between F1 to F8 and MDS-UPDRS-III 3.13 score. A *p* value < 0.05 indicated significant difference for all analysis. As we used multiple tests, *p* value of intergroup comparison among the five different groups were corrected using the Bonferroni method. The alpha value was set at *p’* = 0.05/times of comparison that is *p’* = 0.005. We used ICC to calculate the consistency between the human–machine score. According to previous literature, ICC values less than 0.5 are indicative of poor reliability, values between 0.5 and 0.75 indicate moderate reliability, values between 0.75 and 0.9 indicate good reliability, and values greater than 0.90 indicate excellent reliability [32].

## Results

79 PD patients who completed the data and video collection from October 2018 to January 2020 were analyzed, and 9 of them were excluded due to poor image quality, large clothing and other factors. The postural features of the remaining 70 patients were automatically identified and accurately portrayed. The 70 patients were divided into group of ‘0’, ‘1’, ‘2’, ‘3’ and ‘4’ respectively based on the results of MDS-UPDRS-III 3.13 score. The demographic, clinical data and F1 to F8 were shown in Table 1. A significant difference was found in age (*p* = 0.018), length of disease (*p* = 0.003), Hoehn-Yahr scale (*p* = 0.001), F1 (*p* = 0.010), F2 (*p* = 0.008), F3 (*p* < 0.001), F4 (*p* < 0.001), F5 (*p* < 0.001), F6 (*p* = 0.013), F7 (*p* < 0.001) and F8 (*p* = 0.008) among groups of different MDS-UPDRS-III 3.13 score.

**Table 1:** Demographic and clinical characteristics

MDS-UPDRS-III 3.13 score	0	1	2	3	4	*p*-value
Number	13	12	33	6	6	
Male (%)	6 (46.2)	7 (58.3)	27 (81.8)	5 (83.3)	3 (50.0)	0.084
Age	64.0 ± 6.0	70.3 ± 7.4	69.3 ± 6.3	62.0 ± 11.2	71.3 ± 5.5	0.018^*^
Onset age	61.2 ± 7.0	67.5 ± 8.3	63.1 ± 7.5	57.3 ± 10.8	64.0 ± 7.1	0.170
Duration (years)	2.8 ± 2.8	2.8 ± 2.0	6.2 ± 3.6	4.7 ± 3.3	7.3 ± 5.5	0.003^**^
Hoehn-Yahr	1.5 ± 0.5	1.8 ± 0.8	2.4 ± 0.9	2.7 ± 0.5	2.3 ± 0.5	0.001^**^
F1 (degree)	3.2 ± 4.5	5.8 ± 4.2	9.4 ± 6.3	10.3 ± 12.0	10.3 ± 5.5	0.010^*^
F2 (degree)	0.7 ± 0.9	1.1 ± 1.0	2.3 ± 1.8	4.3 ± 4.8	2.3 ± 1.6	0.008^**^
F3 (degree)	26.5 ± 8.0	34.0 ± 9.9	42.6 ± 9.3	47.8 ± 14.7	54.5 ± 19.0	< 0.001^***^
F4 (degree)	18.7 ± 3.4	20.7 ± 3.4	23.9 ± 4.9	25.3 ± 5.9	41.8 ± 15.1	< 0.001^***^
F5 (degree)	8.4 ± 4.0	9.8 ± 3.1	11.9 ± 5.7	13.2 ± 5.6	25.8 ± 18.9	< 0.001^***^
F6 (degree)	31.1 ± 5.5	31.9 ± 5.0	36.0 ± 6.5	37.7 ± 2.2	39.7 ± 6.5	0.013^*^
F7 (%)	20.2 ± 4.2	22.1 ± 3.3	25.2 ± 4.1	26.8 ± 4.0	46.7 ± 12.9	< 0.001^***^
F8 (degree)	14.2 ± 7.9	19.8 ± 9.0	25.5 ± 9.1	29.2 ± 12.1	20.8 ± 17.0	0.008^**^

*p < 0.05, **p < 0.01, ***p < 0.001

Compared with group ‘0’, post hoc analysis found an increase of F1 (*p* = 0.01) (Fig. 3A), F2 (*p* = 0.027) (Fig. 3B), F3 (*p* < 0.001) (Fig. 3C) and F8 (*p* = 0.01) (Fig. 3H) in group ‘2’, an increase of F3 (*p* < 0.001) (Fig. 3C) and F4 (*p* < 0.001) (Fig. 3C) in group ‘4’. Compared with group ‘1’, post hoc analysis found an increase of F3 (*p* < 0.001) (Fig. 3C), F4 (*p* < 0.001) (Fig. 3D), F5 (*p* < 0.001) (Fig. 3E) and F7 (*p* < 0.001) (Fig. 3G) in group ‘4’. Compared with group ‘2’, post hoc analysis found an increase of F4 (*p* < 0.001) (Fig. 3D), F5 (*p* < 0.001) (Fig. 3E) and F7 (*p* < 0.001) (Fig. 3G) in group ‘4’. Compared with group ‘3’, post hoc analysis found an increase of F4 (*p* < 0.001) (Fig. 3D) and F7 (*p* < 0.001) (Fig. 3G) in group ‘4’.

We further conducted Spearman correlation analysis to explore correlations between F1 to F8 and MDS-UPDRS-III 3.13 score. The results were shown in Table 2, which indicated that there was a significant positive correlation between F1 to F8 and MDS-UPDRS-III 3.13 score.

**Table 2:** Correlation analysis between F1 to F8 and MDS-UPDRS-III 3.13 score

	F1	F2	F3	F4	F5	F6	F7	F8
r_s_	0.388	0.421	0.591	0.561	0.362	0.424	0.603	0.312
*p*-value	0.001	< 0.001	< 0.001	< 0.001	0.002	< 0.001	< 0.001	0.009

r_s_: Spearman correlation coefficient

The grid search and comparative experiments were conducted to search for the optimal combination of input features, decision tree depth and split strategy, which guaranteed the highest human–machine consistency. The selection results were shown in Table 3. The optimal model integrated the input features of F1, F2, F3, F4, F5 and F7, with a max depth of 7 and a split strategy of ‘random’. Under this condition, the ICC between the machine’s and the doctors’ scores was 0.940, which was much higher than the two doctors’ ICC of 0.814.

**Table 3:** Selection results of the relevant features

	**ICC**_**DD**_	**ICC**_**MD**_
F1 + F2 + F3 + F4 + F5 + F6 (max depth = 5, splitter = ‘best’)	0.814	0.707
F1 + F2 + F3 + F4 + F5 + F7 (max depth = 5, splitter = ‘best’)	0.814	0.786
F1 + F2 + F3 + F4 + F5 + F6 + F7 + F8 (max depth = 5, splitter = ‘best’)	0.814	0.781
F1 + F2 + F3 + F4 + F5 + F7 (max depth = 7, splitter = ‘random’)	0.814	0.940

ICC_DD_**:** Intraclass correlation coefficient between two doctors

ICC_MD_**:** Intraclass correlation coefficient between the machine and doctor

According to the optimal decision tree, the feature importance of the selected features to the machine’s automated grading were 13.2%, 12.6%, 16.5%, 11.3%, 6.7% and 40% severally (Table 4).

**Table 4:** Importance of the selected features

	F1	F2	F3	F4	F5	F7
Feature importance	13.2%	12.6%	16.5%	11.3%	6.7%	40.0%

Metrics for evaluating machine learning performance such as accuracy, sensitivity, and specificity were shown in Table 5. Besides, we trained the posture features based on Support Vector Machine (SVM) and k-Nearest Neighbours (kNN) classifiers simultaneously. Their performance did not improve further.

**Table 5:** Classification metrics for the supervised classifiers

	Accuracy (%)	Sensitivity (%)	Specificity (%)
Optimal decision tree	90.0%	89.1	95.7
SVM	88.6	77.4	93.9
kNN (K = 5)	82.9	60.2	90.8

## Discussion

In the present study, we developed an intelligent evaluation system for postural abnormalities in PD based on Kinect and machine learning, enabling the multi-feature, accurate assessment and automated classification. The ICC between the machine’s and doctors’ score was 0.940 (95%CI, 0.905–0.962). All the standard metrics of machine learning performed outstandingly with 90% of accuracy, 89% of sensitivity and 96% of specificity. According to a multicenter clinical trial, the test–retest reliability for the UPDRS-III score which was estimated by ICC was 0.90. ICCs for derived symptom-based scales ranged from 0.69 to 0.88[33]. Therefore, our system is sufficient for clinical application. To our knowledge, this study has the highest human–machine consistency related the automated grading of postural abnormalities in PD.

Ferraris analyzed the posture of 28 PD patients with four features using Kinect [27]. They performed automated machine grading based on three supervised classifier training including kNN, SVM and Multinomial Logistic Regression (MLR). The ICC between the machine’s and the doctor’s grading was 0.74 and the classification accuracies range from 58.8 to 70.8%. Our system developed a new generation with higher accuracy. Taking advantage of a larger data set, we derived more postural features for training the decision tree model. The coronal features (F1 and F2) and the sagittal features (F3, F4, F5 and F7) proved to be very relevant when classifying the PD stage related to posture in the obtained decision tree model. Besides, we applied an objective function for minimizing calculation errors and searching for the optimal results [34, 35]. Both the deviation of the scores and the number of cases with obvious deviation (more than one score of MDS-UPDRS-III 3.13) were considered to ensure that there was less difference between the two scores even in the case where the machine’s and the doctors’ scores were inconsistent. In this way, the accuracy of the automated grading process was significantly improved.

Buongiorno et al. also used machine learning to analyze gait alterations of 14 healthy subjects and 16 PD patients collected by Kinect [28]. The results showed that the Artificial Neural Network (ANN) classifier performed well by reaching 89.4% of accuracy in discriminating PD from healthy control and 95.0% of accuracy in distinguishing mild or moderate patients (two-classes classifier, binary problem). However, it is not yet known how it performed in the finer classification (posture assessment in UPDRS is a five-classification problem). Our system focuses on postural abnormalities and aims to bring an automated and accurate assessment of UPDRS tasks, which can be used for posture assessment before and after medication, enabling dynamic monitoring of postural changes. All these provide effective assistance to doctors and patients. Furthermore, in the follow-up work, we are expanding the application areas of the system. For example, it will be applied to the automated analysis for fine movements of hands and feet, as well as the automated evaluation of sub-item and total score of UPDRS-III. This will bring great benefits to the accurate assessment of PD.

In addition to the above two studies, there are many researchers assessing posture based on Kinect [36–41]. However, machine learning and automated grading are not included, which make it fail to assess the postural abnormality automatically. Moreover, there are also a multitude of technology-based objective measures of postural abnormality that have been developed [14, 42, 43]. Asakawa et al. reviewed how the latest computerized technologies, including the wearable sensors, virtual reality, augmented reality, and robot assistant systems, improved the diagnosis and treatment of PD [14]. However, it is either complicated or expensive of the related equipment [16], which makes them hard to extend the user population and usage scenario. Furthermore, the wearable sensors need to be worn by the patient. Some patients will stand more erect after wearing sensors, which affect the evaluation of the postural abnormalities [44]. Our system is low-cost, non-contact and user-friendly. Patients can get results quickly without wearing any equipment. Besides, the system has simple hardware and software, which is suitable for multiple scenarios such as hospitals, homes and research institutes.

In this work, we found a significant strong correlation between F7 and MDS-UPDRS-III 3.13 (r_s_ = 0.603, *p* < 0.001). F7, which represents the pixel distance between FC and the connecting line of C7 and L5 on the sagittal plane, reflects well the severity of camptocormia in PD patients. Conventionally, three common methods, including total camptocormia method (deviates of C7/L5 line from vertical), upper camptocormia method (flexion between the lower thoracic spine and upper lumbar spine) and lower camptocormia method (flexion of hip) were used to assess camptocormia while they produced camptocormia angles with dramatical difference by up to 50% on the same patient [7]. It requires three parameters to describe the severity of camptocormia accurately. However, to our study, F7 per se accounted for the heaviest feature importance (40%) in the established model, suggesting its key role for machine learning in distinguishing different postural degrees of camptocormia. It is the first time that F7 is proposed and gives a general assessment in patients with both lower and upper camptocormia. Further research shall be conducted to demonstrate whether this single index can serve as a useful and reliable feature to evaluate the severity of camptocormia, especially its relationship between clinical manifestations and life quality of PD patients.

Apart from F7, we also found a significant medium to strong correlation between F3 and MDS-UPDRS-III 3.13 (r_s_ = 0.591, *p* < 0.001) and between F4 and MDS-UPDRS-III 3.13 (r_s_ = 0.561, *p* < 0.001). F3 represents forward flexion angle of head, reflecting the severity of cervical lordosis [5]. F4, which represents total camptocormia angle, was a reliable method proposed by Margraf [7]. Both F3 and F4 are valuable indexes for the severity of camptocormia. Though F6 and F8 were not included in the established decision tree model, it didn’t necessarily mean these two features were not relevant to the severity of postural abnormalities since Spearman correlation analysis revealed both a significant positive correlation between F6 and MDS-UPDRS-III 3.13 (r_s_ = 0.424, *p* < 0.001) and between F8 and MDS-UPDRS-III 3.13(r_s_ = 0.312, *p* = 0.009) respectively. One explanation is that F7 may have covered the content reflected by F6 and F8 in a sense.

The present study has some limitations. Firstly, the number of participants were unevenly distributed among groups of different MDS-UPDRS-III 3.13 score, which may affect the statistical results. Secondly, the posture analysis did not consider the torsion problem, therefore it may underestimate the severity of postural abnormalities in some patients. Thirdly, we only evaluated postural abnormalities of PD patients in the standing state and did not reflect the dynamic changes of abnormal posture while walking. These limitations indicate that the automated assessment of postural abnormalities in PD patients by artificial intelligence needs to be further improved. More optimized algorithms of machine learning, larger data sets and standardized recording modes will continue to improve the detection and accurate assessment of postural abnormalities in patients with complex patterns.

## Conclusions

From our findings, we demonstrate that automated evaluating system, based on the Kinect v2 sensor and machine learning together with the selected extracted features, represents a valid tool to support the assessment of postural abnormalities in PD. F7 was regarded as a key index to evaluate the severity of camptocormia. In addition, the simple and fast feature of the proposed method support and encourage its practicability in a real clinical scenario. Future work will focus on the application of such method on clinical practice, e.g. to evaluate the effect of certain treatments on PD patients and reveal the dynamic changes of abnormal posture.

## Data Availability

The datasets used and/or analyzed during the current study are available from the corresponding author on reasonable request.
